# Correction: Selective capture of hexavalent chromium from an anion-exchange column of metal organic resin–alginic acid composite

**DOI:** 10.1039/c6sc90006b

**Published:** 2016-01-19

**Authors:** Sofia Rapti, Anastasia Pournara, Debajit Sarma, Ioannis T. Papadas, Gerasimos S. Armatas, Athanassios C. Tsipis, Theodore Lazarides, Mercouri G. Kanatzidis, Manolis J. Manos

**Affiliations:** a Department of Chemistry , University of Ioannina , 45110 Ioannina , Greece . Email: emanos@cc.uoi.gr; b Department of Chemistry , Northwestern University , Evanston , IL 60208 , USA; c Department of Materials Science and Technology , University of Crete , 71003 Heraklion , Greece; d Department of Chemistry , Aristotle University of Thessaloniki , 54124 Thessaloniki , Greece

## Abstract

Correction for ‘Selective capture of hexavalent chromium from an anion-exchange column of metal organic resin–alginic acid composite’ by Sofia Rapti *et al.*, *Chem. Sci.*, 2016, DOI: 10.1039/c5sc03732h.



## 

The authors regret that in the original article all values reported with units of ‘L g^–1^’ (5 instances) should have been reported with units of ‘mL g^–1^’. The originally reported values are unaffected by these typographical errors.

The Royal Society of Chemistry apologises for these errors and any consequent inconvenience to authors and readers.

